# Design of a Hybrid (Wired/Wireless) Acquisition Data System for Monitoring of Cultural Heritage Physical Parameters in Smart Cities

**DOI:** 10.3390/s150407246

**Published:** 2015-03-25

**Authors:** Fernando-Juan García Diego, Borja Esteban, Paloma Merello

**Affiliations:** 1 Department of Applied Physics, Universitat Politècnica de València, Av. de los Naranjos s/n, Valencia 46022, Spain; E-Mails: boressan@outlook.com (B.E.); palomamerello@outlook.com (P.M.); 2 Centro de Tecnologías Físicas, Universitat Politècnica de València, Av. de los Naranjos s/n, Valencia 46022, Spain; 3 Instituto Valenciano de Conservación y Restauración de Bienes Culturales (IVC+R), Complejo Socio-Educativo de Penyeta Roja s/n, Castellón 12080, Spain

**Keywords:** cultural heritage, microclimatic monitoring, acquisition data system, wired/wireless, Smart Cities

## Abstract

Preventive conservation represents a working method and combination of techniques which helps in determining and controlling the deterioration process of cultural heritage in order to take the necessary actions before it occurs. It is acknowledged as important, both in terms of preserving and also reducing the cost of future conservation measures. Therefore, long-term monitoring of physical parameters influencing cultural heritage is necessary. In the context of Smart Cities, monitoring of cultural heritage is of interest in order to perform future comparative studies and load information into the cloud that will be useful for the conservation of other heritage sites. In this paper the development of an economical and appropriate acquisition data system combining wired and wireless communication, as well as third party hardware for increased versatility, is presented. The device allows monitoring a complex network of points with high sampling frequency, with wired sensors in a 1-wire bus and a wireless centralized system recording data for monitoring of physical parameters, as well as the future possibility of attaching an alarm system or sending data over the Internet. This has been possible with the development of three board’s designs and more than 5000 algorithm lines. System tests have shown an adequate system operation.

## 1. Introduction

Preventive conservation methods are based on the concept that controlling some of the major deterioration causes in the environment it is possible to ensure the sustainability and durability of the art work [1].

Depending on the nature of cultural heritage, the deterioration causes are subjected to the influence of different physical parameters. For example, artworks are influenced by terms of stress caused by physical agents such as temperature, humidity, radiation, and chemical agents (e.g., CO_2_, SO_2_, O_3_, mineral salts, *etc.*) [2], meanwhile, temperature and humidity can reach especial importance on archaeological structures because of being majorly stone-built [3,4].

But generally, abrupt changes of temperature and relative humidity (RH) may cause serious damage in all the different kind of objects, such as non-isotropic material deformation or detachment in materials of several layers [5]. In hygroscopic materials, such as wood panels, which are the mainstay of many artworks, mechanical changes and deformations could occur [6]. In the case of frescoes, soluble salts and moisture are the very common causes of deterioration; therefore an early detection of dangerous values of these physical parameters (together with other chemical parameters in some cases) is essential to avoid this kind of damage [7].

Thus, both long-term monitoring of the environmental parameters as well as further analysis of the recorded data are necessary [8]. The concept of “Smart City” is notoriously liquid, scarcely formalized and, in some degree, subject to different ideological interpretations [9]. Normally Smart City is associated with real-time monitoring and cloud computing [10]. However, Smart Cities should not necessarily be restricted to real-time monitoring. In fact, in the case of monitoring cultural heritage, historical data (without underestimating real-time alarms, *etc.*) is more interesting as it is important to characterise the site for performing comparative studies in the future, either regularly, in order to verify whether the conditions are constant, or occasionally, when the boundary conditions are altered [11].

Thus, the aim of monitoring cultural heritage in a Smart City would be sending all these recorded data to internet so they can be checked and serve as an example for the conservation of other similar sites.

The environmental monitoring is performed by a regular data recording, whose frequencies typically range between one datum every hour (1 datum/h) or every day (1 datum/day) [12,13].

Monitoring with frequencies near 60 data per hour (1 datum/min) is interesting as power and confidence of the statistical analysis performed increases in direct proportion to the sample size used [8], and therefore develop a monitoring system with large grid of sensors and big memory to store large amounts of data from high recording frequencies is interesting.

Different works monitoring cultural heritage are found in the literature [14,15]. Most of them use individual data-loggers. Some authors [16] use Hobo data-loggers [17], and other studies [6,11,18] work with data-loggers DS1922L [19] and DS1923 [20]. Data from these data-loggers cannot be sent automatically to the cloud and due to they are commercial devices, its hardware and software is unknown and cannot be modified.

There are also papers that have studied the microclimate in churches making use of a wired sensor network composed of different lines and sensors and a single microcontroller [7,21]. The experience with the DS1923 [18] has demonstrated that individual data-loggers battery and memory get depleted around the third month of work in cases where data has to be taken with high frequency. Moreover, results of each data-logger must be combined into one file. Software to combine the data automatically once they are in the computer could be developed; however the hardest part of the work consists in loading data into the computer one by one.

A sensor network solves the issue of the lack of memory and battery but only SDI-12 [22] protocol is able to reach cable length of 250 m and devices with this protocol are much more expensive and do not allow connecting enough sensors (no more than 60) to cover cultural heritage applications [21].

Therefore, the best option is to develop a new kind of network data-logger adding more cable length, amount of sensors connection, flexibility and lower consumption and price than a computer and other data acquisition systems.

The own development of monitoring systems from the experience of the requirements of the particular field of action is essential for the technical and cost efficiency of these [23]. Some papers have developed acquisition systems for remote control applied to cultural heritage [24,25].

This paper deals with the necessity of a low cost data-logger able to collect and record data of physical parameters from extended areas for preventive conservation applications, combining wired and wireless communication protocols. In this paper we present a device able to communicate with sensors using 1-Wire protocol, programmed to control the frequency of data capture, with a failure protection system, meeting as much as possible with the requirements of the market and consumers and with a versatile programming that allows its extension to different application such as alarms, sending data via Internet, *etc.* for Smart Cities.

## 2. Materials and Methods

### 2.1. Sensors

The device will be developed thinking on the sensors selected and their protocol of communication. In this case, sensors are predetermined by Maxim Integrated Company for its proven operability in cultural heritage purposes [7,21], as well as its wide range offer of sensors, analogue to digital converters and not expensive prices.

A practical application for the testing of the system is performed at the end of this paper. Two 1-Wire slaves were assembled in a single double sided printed board of 12 mm × 26 mm: a DS18B20 [26], which is a temperature sensor with an accuracy of ±0.5 °C, with a range of measurements from −10 to +85 °C, and an Analog to Digital Converter (ADC) DS2450 (Maxim Integrated Products, Inc., San Jose, CA, USA) [27] which can use one of its four channels to read the analog potential of a HIH 4030-001 RH sensor [28] (Honeywell International, Inc., Minneapolis, MN, USA) with ±3.5% RH accuracy (uncalibrated) and a range of measurements from 0% to 100% of relative humidity.

The Maxim Integrated devices have their own and unique serial number implemented in production and impossible to change. A calibration procedure with aqueous solutions of two salts (lithium chloride and sodium chloride) was applied in laboratory according to the standard ASTM E 104–02 [29] in order to study the measurement errors of RH sensors. All RH sensors were introduced in a small chamber equilibrated with a saturated solution of a salt. Next, calibration equations were obtained for each sensor in order to relate the voltage output and RH.

### 2.2. Wired Transmission Protocols

The protocol selected for controlling the sensors by the master is 1-Wire. The number of sensors and the restrictions to install them in each application are quite unpredictable. So to offer a major flexibility into the data logging network, the structure of the data-logger is going to be divided into three different modules (Master module, Slave module, and Wireless module). In order to make available the communication, each module has to have its own microcontroller, so one is installed in each module, and I^2^C protocol is selected to communicate modules physically connected. This protocol will be used for the data transmission through the board as it offers a good baud rate with synchronic communication and it is very simple to implement.

### 2.3. Wireless Transmission Protocol

The system includes an xBee module to offer wireless support [30]. This device is able to route messages using the IEEE 802.15.4 standards. This protocol offers to these modules the next features: low tax of noise interference, low tax of interferences, low power consumption (depends on the actuation range), high taxes of transmission power and reception sensibility, extra encryption services (application and network keys implement extra 128b AES encryption), association and authentication (only valid nodes can join to the network), AODV routing protocol [30]. This allows the data-logger to send information about its sensors to a Master device in order to store data. Furthermore, the system can include as many wireless data-loggers as is necessary to accomplish the number of sensors and distance requirements.

### 2.4. Microcontroller ATmega328

In order to dispose a simple programming environment all the libraries were implemented in C++ for the microcontroller. The company Arduino offers a solution with all the microcontroller functions needed for the application. Specifically microcontroller ATmega328 is available in the market with a low price and has “Arduino UNO” board to program the microcontroller.

The ATmega328 has the minimal requirements for the application. It has one I^2^C port in order to connect with the device DS2482-800 [31] and the external clock [32] together with an USART to connect other peripherals like an USB memory. The programmable memory is 32 kB so it is enough to store all the necessary libraries, the serial number of the sensors and the main program. Moreover, it is equipped with digital and analogic inputs and outputs modulated by PWM to improve or adapt our data-logger to a wide range of possibilities.

Communication between microcontrollers is possible using the I^2^C port between modules and the USART port for the wireless device. Arduino Integrated Development Environment (IDE) provides all the libraries to enable this communication. As the microcontroller does not have 1-Wire port, device DS2482-800 of Maxim Integrated [31] manages automatically 1-Wire protocol enabling the system to connect a theoretically unlimited number of sensors per channel and a kilometre of wire. This device is equipped with 8 different 1-Wire channels.

### 2.5. External Clock

The DS1307 (Maxim Integrated, Inc., San Jose, CA, USA) is the device implemented as external clock on the printed circuit board. This device also uses I^2^C protocol to communicate with the microcontroller, has a low consumption energy rate and an external battery for guaranteeing the operation if the main input current falls down [32].

### 2.6. External USB Memory

Data-logger is developed to save all data inside an external memory by the use of a peripheral USB.

The device used to arrange the USB is the peripheral VDIP1. This device and its microcontroller FTDI have been developed by the company Fdichip [33] and include all the necessary programs installed to provide access by the UART, parallel FIFO or SPI interface pins on the main microcontroller. The communication selection is provided by two jumper pin headers to allow by simple configuration of the I/O on data and control bus pins of the VDIP1. In this case, the communication is managed by the UART port connected to the USART port of the main microcontroller.

### 2.7. DC Power Supply

Devices NCP7805TG [34] and UA78M33CKCS [35] have been installed on the board in order to offer a stable output current with a high rate of input voltage.

### 2.8. The Failure Management

Failure management system includes the aspects and devices that protect the data acquisition against unexpected events. To protect the system, the data-logger will include a device named “Watch-Dog”, specifically the TPS3813 by Texas Instruments [36]. This chip is used to reset the complete system (without deleting any data) in order to restart the configuration into a known working point. This test is performed in time intervals that will be set by the developer according to the sampling frequency and the number of sensors.

Furthermore, Time Outs in all the loops to resume the program in case of fails in the communication protocols and data acquisition will be included in the program. The last of these Time Outs can be modified in the main program by software in order to improve the communication depending on the amount of slaves in the system.

### 2.9. Description of the Testing Experiment

An experiment of monitoring was performed in order to check the system operation, without the goal of diagnosing if the environment surrounding the selected artwork is suitable for its conservation. Results obtained with the developed system are compared with those of a commercial data-logger that has been used before in monitoring of cultural heritage.

Each probe of these autonomous devices consists of one temperature data-logger (model Thermochron DS1922L [19]) and one RH data-logger (model Hygrochron DS1923 [20]). Each data-logger (DS1923) contains a humidity sensor with an accuracy of ±5% and a temperature sensor with an accuracy of ±0.5 °C. RH data-loggers were calibrated with aqueous salt solutions (as done with sensors in Section 2.1) according to the standard ASTM E 104–02 [29].

In 1928, the textile industrialist Eduardo Romero Sanchis placed a facsimile, with detail and identical to the Real Senyera on order of the City Council of Valencia, weaved in silk: four strips of magenta silk on golden silk tissue background, with a blue stripe on the flagpole side, with a golden crown, also in golden tissue [37].

When the worldwide recognized Valencian writer Vicente Blasco Ibañez died in 1928, the flag was moved to France to cover her coffin. Some years after, in 1932, it was moved for the same purpose during his funeral in Valencia after the transfer of his remains.

**Figure 1:**
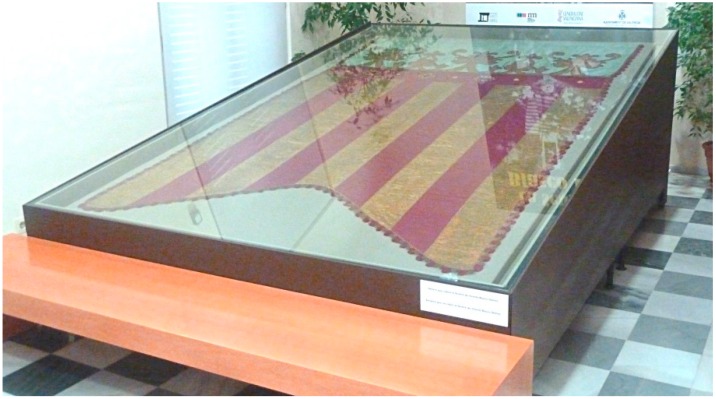
Restored flag in the display cabinet, Blasco Ibáñez Museum-House (Valencia).

In December 2009, the political party *Union Valenciana* donated the flag to the Valencian Government under the condition of its restoration and conservation. Currently it is displayed, after its restoration in 2013, in the Blasco Ibáñez Museum-House (Valencia, Spain), inside a sealed slightly inclined display cabinet (317 × 223 × 12 cm) designed for this purpose (Figure 1).

A pair of sets of two DS2450 and HIH-4030 sensor probes were installed (a couple inside the display cabinet and the other at the outside), and probe set with one commercial DS1923 data-logger was installed next to the sensor at the outdoors of the display cabinet as a testing data-logger.

For the experiment, sensors were installed indoors and outdoors the display cabinet. Our developed monitoring system is composed of three different modules (Master module, Slave module, and Wireless module that will be described in Section 3). In the outdoors of the display cabinet, the Master and a Slave managing a line of two sensors (one inside and the other outside the display cabinet) were placed. The monitoring experiment started on 17 November 2014 and ended on 27 November 2014. During this period of 11 days, each commercial data-logger recorded one measurement every 10 min (6 data per hour) and each data-logger of the designed system recorded one measurement every minute (60 data per hour).

## 3. Design of Hardware and Software for the Data Monitoring System

The structure of the data-logger is going to be divided into three different modules (Master module, Slave module, and Wireless module). The goal of the Master module is to collect data from all the slave modules installed in the wireless network and manage them in order to save the information inside a USB memory (in this project) or sending the information to other device such as a computer.

The Slave module is the responsible for collecting data from the sensors and sending the information to the connected module.

The Wireless module is able to send the information received from the connected module to other Wireless module or the opposite.

It is designed to connect two modules and form three different kinds of data-loggers. For example if we connect a Master module with a Slave module we will have a common data-logger, but we can create a wireless network system using two Slave modules and a Master module connecting each one with a Wireless module. That way, two different data-loggers can collect data from two different places and save them into the same external memory by wireless, allowing one, for example, to easily monitor different rooms in a museum with a unique monitoring system.

### 3.1. Design of Hardware for the Data Monitoring System

The first subject is the printed circuit board (PCB) design because is the basis of the production. For the development of this Section the main program used for the designs is “Eagle CAD” [38]. When we started there is not supposed to be a limit of size but it is recommendable to minimise it in order to offer a good design. Furthermore, the free version of “Eagle CAD” only allows a certain maximum size of (56 × 36 × 23 cm). During the design, a prototype had been created in order to solve possible troubles during the construction and the working life. The final solution is presented in this Section.

#### 3.1.1. Design of the Power Track

Two different supplying systems are included. One +5 V DC current for the alimentation of the ATMega328 and others. This alimentation will be installed on the Master and Slave modules. One +3.3 V DC current for the alimentation of the xBee integrated (Wireless module).

The selected devices to supply the power are the NCP7805TG regulator for the +5 V and the UA78M33CKCS for +3.3 V, allowing the data-logger being connected to a wide range of DC transformers.

Besides the power filter has been improved from the Arduino design, adding a small ceramic capacitor, using parallel connexion, to the electrolytic filter. Thus, final combined filter has better proprieties in terms of resistance and price.

Note that, if the location has a high rate of illumination, it could be considered the option of looking for a position with enough luminal intensity in order to include a photovoltaic source to recharge a data-logger battery. Once the supply system has been designed, the next step is to define the position of each component and connect the tracks with the external connections. This step will be different in all the modules.

The PINs used to connect different boards have to cover both sides in order to be able to have a module up and also down. The connection distribution is similar to the Arduino modules, that way it is possible to create new applications just adding new software and the corresponding board. A reset switch is also available in the Master and Slave modules; moreover this signal affects the connected Wireless module too.

#### 3.1.2. Final Boards and Schematics

**Figure 2:**
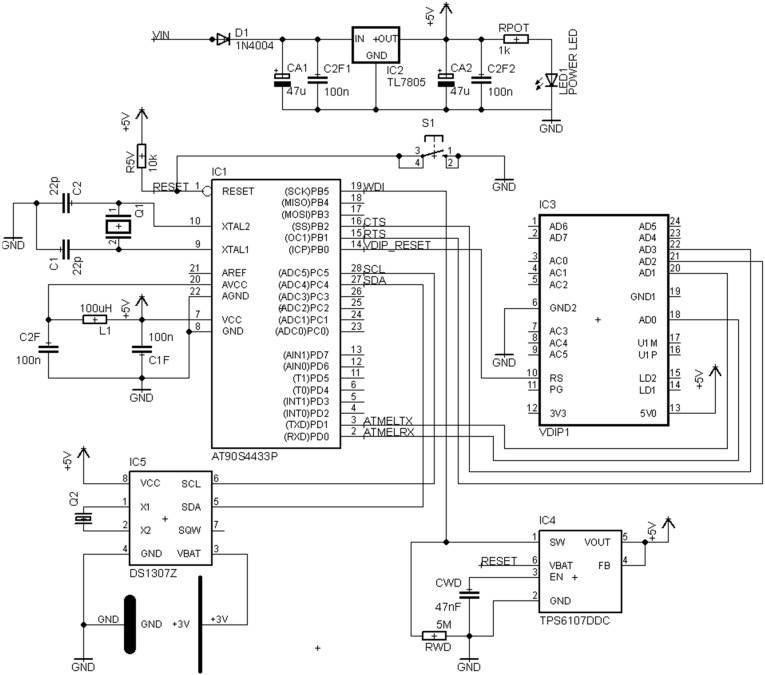
Master schematic final design.

The majority of the power tracks such as GND, +3.3 V and +5 V are designed with 0.8128 mm of width. The rest of tracks, as far as is possible, are about 0.5 mm. In the final design, depending on the module, the connected devices are different. In the Master, the VDIP1 is connected with the ATMega328 using UART port (Figure 2). Besides, to ensure a correct communication between them, two of the ATMega328 PINs are used as “Clear to Send” (CTS) and “Request to Send” (RTS) (Figure 2). Those tracks manage when both are ready to enable a data transmission.

On the other hand, the DS1307 (External Clock) has been connected to the PINs ADC5 and ADC4 of the ATMEGA328 using “Serial Data Line” (SDA) and “Serial Clock Line” (SCL) in order to enable the I^2^C communication between them. Regarding the Watch-Dog, the “Watchdog timer input” (WDI) has been connected to one of the PINs of the ATMega328 to enable the Failure Management.

Finally, an optional port has been installed to manage a “liquid crystal display” (LCD), which could be installed in the prototype to ensure correct data acquisition.

**Figure 3:**
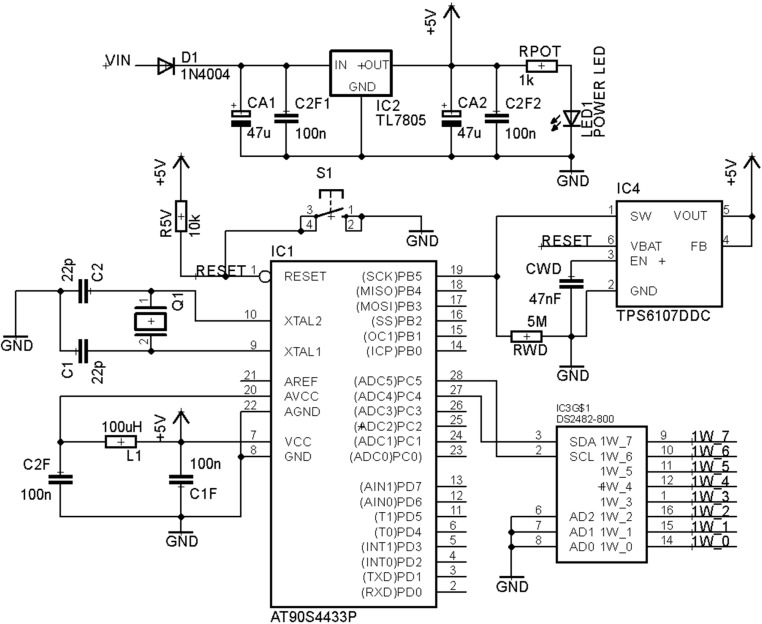
Slave Schematic Final Design.

Secondly, on the Slave module, the USB peripheral and the external clock have been replaced by the I^2^C to 1-Wire translator (Figure 2 and Figure 3). The external 1-Wire plug is the standard D-sub connector MHDD which offers 9 connexions: 1 to the Ground (GND), 1 to the Sensors Voltage supply (VDD) and 7 channels. The channel number 3 output is reserved and located inside the board in order to offer possible connexions inside the encapsulation like other kind of sensors or protections.

**Figure 4:**
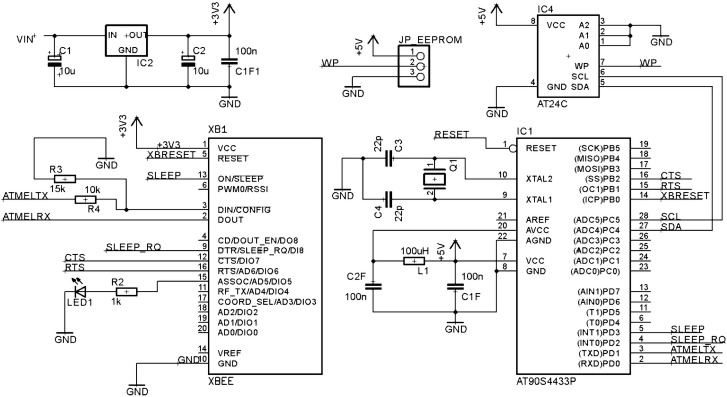
Wireless Schematic Final Design.

The Wireless module has an XBee microcontroller (Figure 4) to enable the wireless communication between two different modules by wireless (able to communicate among them using the IEEE 802.15.4 protocol and communicate with the AtMega328 using the USART port). It can be used to communicate also Master or Slave modules or a commercial Arduino board by I^2^C making it very versatile and useful in a wide range of applications.

To avoid interference between the module which is connected (for example a Master module and its LCD connection) the module only has connected to the Digital Arduino connexions those necessaries for the communication with the attached module (PINs D2 and D3). However it is not the case of the analogic connexions (A0, A1, A2 and A3), so if those inputs are going to be used during a new designing process, it is necessary to break the connexion between the connected modules (it must be taken into a count that the channels A5 and A4 are the “SCL” and “SDA” I^2^C signals so their connexion never should be broken).

**Figure 5:**
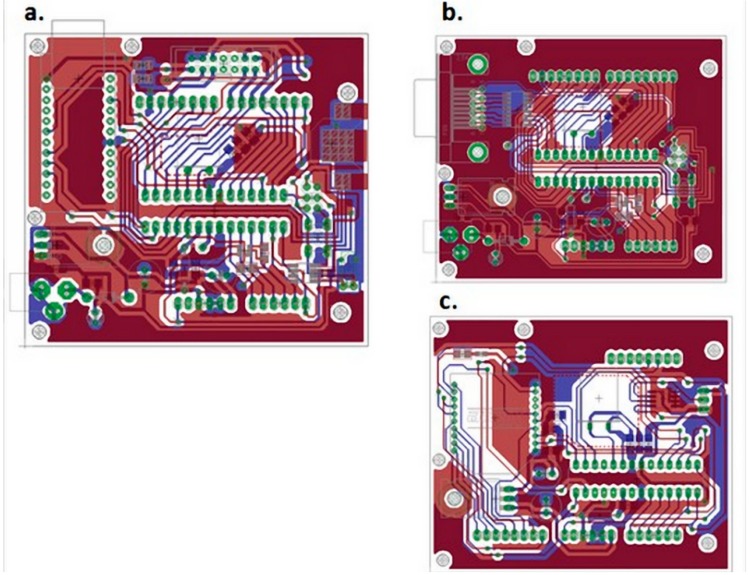
(**a**) Master PCB Final Design; (**b**) Slave PCB Final Design; (**c**) Wireless PCB Final Design.

Moreover, to make the most of this module, an EEPROM memory has been installed on the top of the board and its data is protected by an external jumper. The final PCB boards of the three designed modules, compatible with Arduino module, are shown in Figure 5.

#### 3.1.3. External Clock

An external clock has been included in the printed circuit board. This clock can use a button battery which allows it continue counting even when the complete system is not connected to an energy source along more than a year. That way it is only necessary to set the clock on time once a year during the data-logger revision.

Besides, the time accuracy of external clocks is usually more precise than microcontrollers in long periods. The microcontroller is continuously recording the time data and it is saved in the EEPROM memory. This aspect is really useful to recover the data time if the external clock stops.

#### 3.1.4. External USB Memory

In order to simplify the data storage and the final data recording, the data-logger is developed to save all data inside an external memory by the use of a peripheral USB. This memory has to be connected along all the process of data-logging, therefore if there is not a USB memory connected, the data-logger will be unable to save data and the data acquisition will be interrupted.

In case of a current failure, or the destruction of the microcontroller, it is really easy to recover the data extracted before the breakdown. Also, this technique avoids the necessity of developing a procedure to transmit all the data to a computer or to another USB-PENDRIVE. When we extract data there is no need to turn off the data-logger. A red LED is on when the process of saving data is taking place.

The requirements of the external memory are not too expensive. All kinds and brands of USB Memories are allowed. Even low level memories around 100 Mb ensure the autonomy of the data-logger for more than one year recording data of 50 sensors and one data per minute.

### 3.2. Design of Software for the Data Monitoring

Computer language C++ [39] has been used for the development of all algorithm programs. All the programs are designed using Arduino commands and Wire library (I^2^C communication) [40]. In this Section we detail libraries created especially for the data-logger.

#### 3.2.1. Control Algorithms of Modules

Algorithms used to provide to the main programs inside the ATMega328 the necessary tools to develop an efficient control of the data-logger have been developed. In order to provide of versatility the board and security to critical variables, all the libraries have been designed as “Objects” so they have a “Constructor” and a “Destructor” which have to be managed by the main program.

As control algorithms four libraries have been developed (VDIP1ART, DS1307ART, TPS3813ART and SENSORSART). VDIP1ART provides a range of functions which ensure a correct communication between the VDIP1 device and the microcontroller in order to write a message inside an USB EEPROM memory. DS1307ART provides the necessary tools to read and modify the hour and the date of the External Clock (DS1307). Furthermore, it has a function to ensure that the clock is working correctly. The functions inside the TPS3813ART are designed to offer support to the Watch-Dog device. In this case, the Watch-Dog is running since the power supply starts up, there is no need of a “Constructor” to initialize the device, but it is important to restart the countdown of the reset. The reset countdown is controlled by the discharge of a capacitor situated close to the device, if the discharge is complete, a reset occurs. The WDI track is the responsible for the capacitor reloading, but if there is too much time between WDI activations, the capacitor is discharged so the microcontroller reset will be done.

SENSORSART library is an ensemble of subclasses assembled to offer support to different kinds of sensors using the controllers of the DS2382-800. Each kind of sensor has its own initialization and as it is an object, it is also possible to define different global calibrations to different sensors (even being the same type). Besides, it provides special functions in a subclass “DSSearch” to extract the serial numbers of the sensors connected to the 1-Wire.

The program structure of all modules is lineal. The Master module program follows the next structure: constructs the objects, initializes the parameters, waits the next minute, requests data, opens the file, writes the date, and closes the file. To accomplish this aim, the Master needs the VDIP1ART (to store the data inside the USB memory), the DS1307ART (to evaluate the moment of extracting data) and the TPS3813ART (for the failure management).

Depending on the application, data could be organised on different ways inside the USB memory. In this application the number of the sensor is indicated by the relative position in the line. When there is a problem collecting data from one channel, the Master writes null values at the position of these sensors.

The program structure of the Slave module is the following: constructs the objects, initializes the parameters, reads the sensors data, stores them in the EEPROM memory, waits Master module order, sends data. To accomplish this aim, the Slave needs the SENSORSART (to read sensors data).

The program structure of the Wireless module is also lineal: constructs the objects, initializes the parameters, waits an order, interprets the order, sends the order. As this module has two communication channels, wireless and I^2^C channel, the answer is never sent using the channel of receipt. The Wireless does not use any of the previous developed libraries; it only uses Wire library included in Arduino’s IDE.

A Cyclic Redundancy Check (CRC) is implanted inside 1-Wire and I^2^C communication protocols, between the sensors and the Master module in order to ensure that there has not been any mistake in the communication. An example could be the data collision by the response of two slaves at the same time or interferences.

Using this data protection, it is possible to trust with a high level of trustworthiness that the bytes read by the Master are the same that the ones sent by the slaves [41]. So in case of a data error, it must be a problem with the sensor, but not with the transmitted information. The CRC cannot correct data, but is the most efficient way and with the highest probability to detect mistakes in the messages.

In this case the CRC analysis is implemented by software in order to simplify the printed circuit board but at the cost of increasing the complication of the program code. That is because the speed is not an important factor in the design.

#### 3.2.2. Communication Protocols

For a better understanding of the communications, Table 1 shows a glossary of all symbols used in the flowcharts and communication examples.

**Table 1:** Glossary of Symbolism used.

Symbol	Description
akn	Acknowledge byte
Buff_S	Serial buffer
Ch	Sensors Channel
CRC	Cyclic Redundancy Check
DP	Data Positions (2 bytes: [First_Byte_Position, Last_Byte_Position])
H_Ch	Channel header for the transmission of the selected channel
H_Dt	Data header for the transmission of the Data from Sensors
H_FDt	Data header for the transmission of the last message of Data from Sensors
LDP	Last sensor data
M_Dir	Wireless Module Address
MSign	“*Sign*” always checked as correct coming from a Master Wireless
Nakn	Negative Acknowledge byte
Ncrc	Negative Cyclic Redundancy Check Validation
O_CCmd	Order of Canceling Command, also used at the end of an instruction
O_MRDt	Order from Master to Request Data from his Wireless Module
O_MRMd	Order from Master to Request Data from a Module of Sensors
O_SRCh	Order from Slave to Request the channel to read the Data from Sensors
O_SSDt	Order from Slave to Save the Data from Sensors
O_STDt	Order from Slave to Transmit the Data from Sensors
O_WRDt	Order from Wireless to Request Data from a Channel of Sensors
O_WSDt	Order from Wireless to Save Data from a Channel of Sensors
O_WTDt	Order from Wireless to Transmit the Data from Sensors
PIN_B	Send Buffer PIN
PIN_R	Request PIN
RFrom	Request From
Sign	Validation bytes for the transmission of Data
XXSD	Information Bytes (XX means de number of bytes)

### 3.3. Communication between Wireless Modules

The Wireless module algorithm is always the same, but its behaviour depends on the module to which it is connected. When the connected module request data to the Wireless module, it uses the I^2^C protocol. On the other hand, communication between two Wireless modules uses Serial port. When a communication is requested, the Wireless module analyse the message depending on if the message comes from Serial (from other Wireless module) or from I^2^C (from a Master or Slave module). On the first case, the message has to comply with the standards and, if the message is correct, the Wireless module will accept the order and send an *akn*, otherwise it will ignore the order and send *Nakn* or a *Ncrc* if any byte has been corrupted during the transmission.

**Figure 6:**
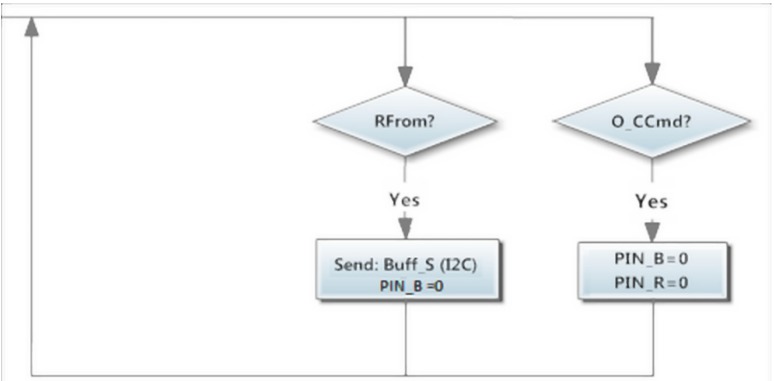
Wireless module, I^2^C Support Orders.

In order to offer support to the I^2^C communication, two digital pins are used on the Wireless module, *PIN_R* and *PIN_B*. *PIN_B* = 1 indicates that a message is available to be read from the exit buffer of the wireless so the Slave or the Master module can send a *RFrom* command (Figure 6) to read the message. Meanwhile, *PIN_R* = 1 indicates that the last command order has not been performed yet.

**Figure 7:**
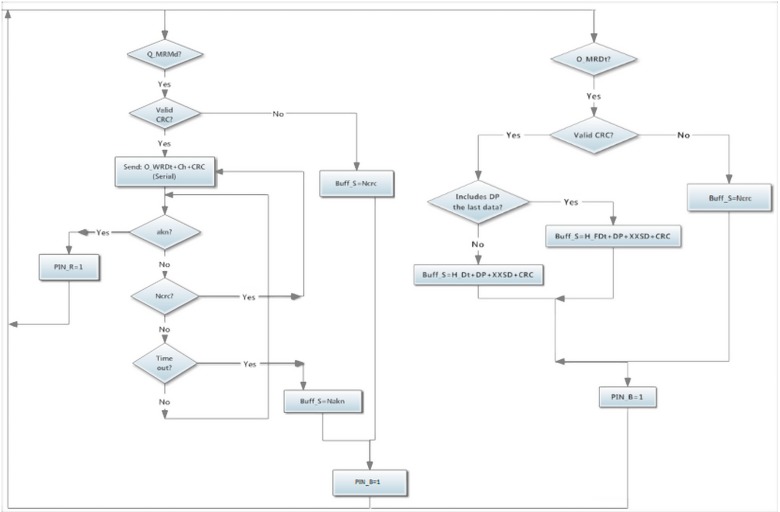
Flowchart of wireless orders from Master module.

Finally, an *O_CCmd* (Figure 6) can be used to put both *PIN_R* = 0 and *PIN_B* = 0, interrupting any communication state. Data transmission always starts with the Master sending a request by I^2^C to its Wireless module using the *O_MRMd* command (Figure 7). When the information is read, it contacts to the correct module to request its data using the *O_WRDt* command together with the value of the desired sensors channel.

**Figure 8:**
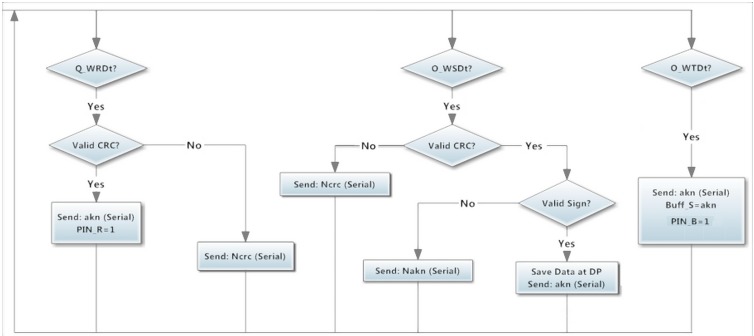
Flowchart of wireless orders from Wireless module.

When a Wireless module has been contacted by *O_WRDt* command (Figure 8), it sends an interruption using *PIN_R* = 1 indicating to the Slave module that a data request has been managed. Then, a data transmission starts in order to load the sensors data from the requested channel.

Once the requested module has loaded the information from the Slave module, it starts the Wireless data transmission. All sensors data are sent in defined transmission messages to the receptor data Wireless module. This action is performed using the *O_WSDt* command (Figure 8) which requests to the receptor data Wireless module the action of storing the sensor readings on the indicated *DP* memory positions. In order to do not overflow the *Buff_S*, the messages have a maximum of 10 sensors values (buffer size is 64 bytes).

In each message, the *Sign* bytes identify where the data are coming from. The Wireless module connected to the Master module has to decide if those data are as expected. If the data are accepted it will send an *akn* byte, otherwise a *Nakn* byte will stop the sending data emitter.

If the transmission is completely accepted and the emitter has sent all its data, the *O_WTDt* command (Figure 8) will indicate to the receiver that the transmission has ended and, therefore, it can start its communication with the Master module.

### 3.4. Master Module to Wireless Module Communication

The Master-Wireless communication starts with an *O_MRMd* command (Figure 7) followed by a *O_MRDt* command (as shown in *Wireless module communication Section)* together with the *M_Dir* and desired *Ch*. *PIN_R* = 1 means the action is being performed. When the Wireless connected to the Master has the data from the sensors of the requested module, it sends an *akn* to the Master module in order to indicate that the transmission has been successfully performed.

After this, the Master sends an *O_MRDt* command to the Wireless module to indicate the desired sensors with the *DP* bytes. Once the Wireless module has those data inside the exit buffer, it sets *PIN_B* to indicate the data are ready for a *RFrom*. This communication has a byte that indicates which kind of data is sent as header, for example, if the *DP* includes the data from the last sensor, the header will indicate it. When the transmission has finished and the Master has all the data, it sends an *O_CCmd* (Figure 6) command to release the Wireless module so it sets *PIN_R* = 0 again.

### 3.5 Slave Module to Wireless Module Communication

The Slave module algorithm is designed to collect data from all sensors connected while the Master module does not request communication. That way, the state of its sensors is always updated. This method of keeping all data updated is useful for future applications as the Slave module can control in real-time the state of the wired network of sensors. Thus, the slave can start a communication with the Master module through the Wireless to activate, for example, an alarm.

**Figure 9:**
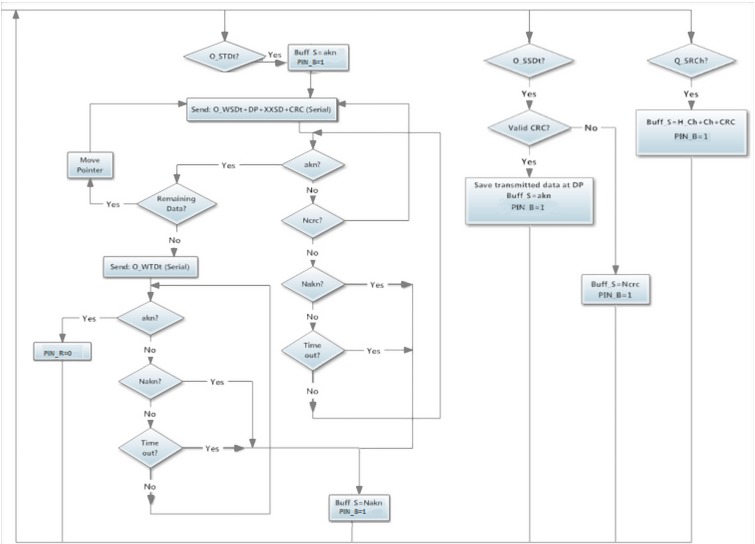
Flowchart of wireless orders from Slave module.

When the Wireless module to which it is connected indicates that a data transmission has been requested *PIN_R* = 1, so an interrupt inside the microcontroller occurs and the Wireless module starts the data transmission using the I^2^C protocol. The Slave module asks for the requested channel sending an *O_SRCh* command (Figure 9) to the Wireless slave module, then, it loads into its I^2^C exit buffer the number of the channel requested by the Master Module and indicates that the message is available to be read. Therefore, the Slave module sends a *RFrom* command to read the message with the requested channel.

After this, the Slave module, using the *O_SSDt* command (Figure 9), sends the sensors stored data and the Wireless stores this data inside the indicated *DP*. When the Slave module has finished, it sends an *O_STDt* command with the number of *LDP* in order to indicate that the Wireless module can start the transmission with the emitter of the request. If the transmission between Wireless modules has been accepted *PIN_R* = 0, indicating that the transmission has been performed correctly.

**Figure 10:**
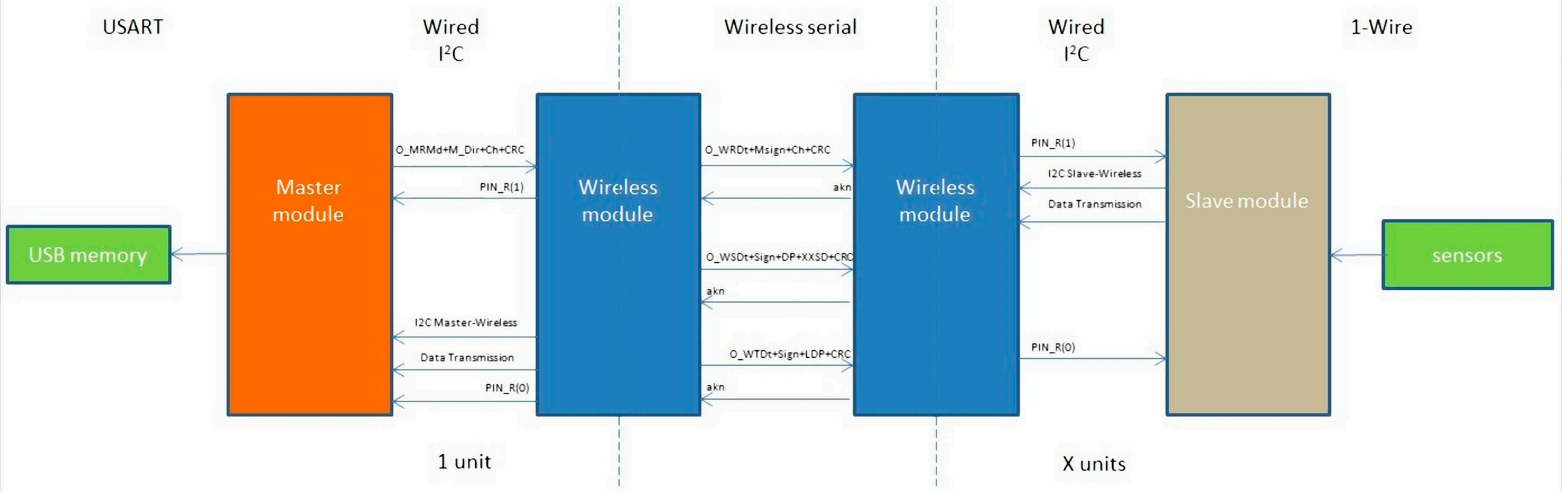
Global communication example.

The communications protocols between one Master and one Slave are shown in Figure 10 in simplified as an example; note that a wireless network could have more Slave modules.

### 3.5. System Testing

**Figure 11:**
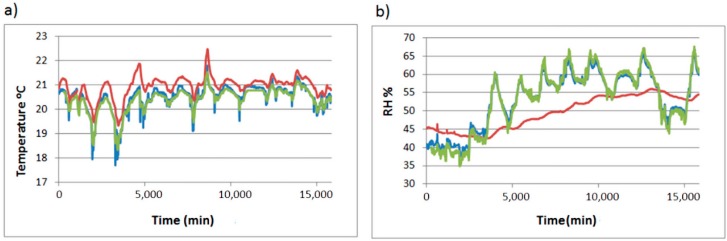
Trajectories of sensor inside the display cabinet (red), sensor at the outside of the display cabinet (blue) and control data-logger at the outside of the display cabinet (green). (**a**) Temperature; (**b**) RH.

The developed software has been monitoring for a period of 11 days, with a monitoring frequency of one datum per minute, obtaining a total of 15,840 datapoints per sensor (60 data/h × 24 h/day × 11 days). Furthermore, commercial data-loggers recorded data every 10 min (for the control of the developed system). The trajectories of the three sensors are shown in Figure 11.

**Figure 12:**
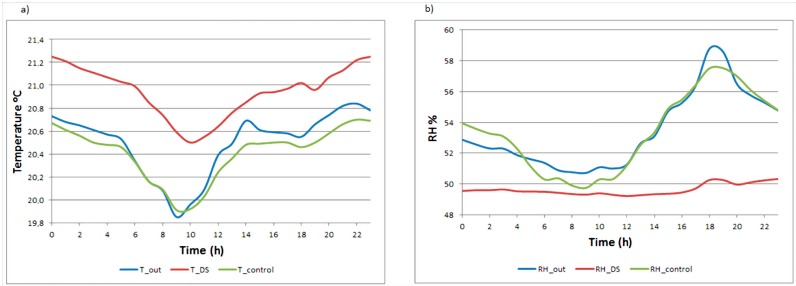
Mean daily trajectories of sensor inside the display cabinet (red), sensor at the outside of the display cabinet (blue) and control data-logger at the outside of the display cabinet (green). (**a**) Temperature; (**b**) RH.

The mean daily trajectory of data recorded inside the display cabinet and outside of it, as well as data recorded by the commercial data-loggers have been plotted to assess the results obtained by our monitoring system (Figure 12).

Each RH measured by our system out of the display cabinet differs 1.09% on average from the control data-logger. For the temperature the difference is 0.46%. In absolute terms, this mean difference is 3.72% RH and 0.54 °C for temperature. These values are within the error range of the sensors (±5% RH and ±0.5 °C).

## 4. Conclusions

A device which accomplishes the main requirements for being used in monitoring physical parameters for cultural heritage purposes has been successfully developed. One Master, one Slave and two Wireless prototypes modules have been designed to verify the consistence of the designs.

The modules can be connected conveniently to configure a monitoring system that suits the cultural heritage site. In addition, third party hardware is used in order that the system can be very versatile because different modules available on the market can be attached for sending an alarm by SMS, recording data in the cloud, switch on/off an HVAC system, *etc.*

As the system reads a four channel ADC (DS2450), each integrated circuit can connect up to four sensors that transform the measured physical parameter into a change in the electric potential.

Libraries have been programmed for a failure protection system using a Watch-Dog and identifying corrupted bytes inside data transmission. Thereby, it is ensured that stored data is correct and the system does not come into infinite loops or crash. Furthermore, an internal clock that controls the frequency of data capture has been programmed.

Moreover, due to the time it takes to save every data, the prototypes have been able to save the measures from 80 sensors extracted from the Slave module inside a USB memory in less than 30 s of time and record measures of temperature and humidity from a surface of 2000 m^2^.

The system has a data storage capacity which depends on the USB memory used. For a 2 GB USB memory and 20 sensors recording data every minute the system will be able to save data continuously during 10 years. Even low level memories around 100 Mb ensure the autonomy of the data-logger for more than one year.

The whole system has been made using free hardware-software or trial versions so its development has been possible thanks to the open scientific knowledge on the Internet. A practical testing of the system has revealed its suitability and matching results with commercial data-loggers used in cultural heritage.
